# Assessment of Borderline Personality Disorder in Geriatric Institutions

**DOI:** 10.3389/fpsyg.2021.629571

**Published:** 2021-03-23

**Authors:** Franck Rexand-Galais, Lucas Pithon, Johane Le Goff

**Affiliations:** ^1^BePsyLab and Spaces and Societies Research Team, Department of Psychology, University of Angers, Angers, France; ^2^Korian Bollée-Chanzy Nursing Home, Le Mans, France; ^3^St Vincent de Paul Nursing Home, Yvré-L'Évêque, France; ^4^BePsyLab, University of Angers, Angers, France; ^5^Gerontological Center of Lucien Hussel Hospital Center, Vienne, France; ^6^Research Center in Psychopathology and Clinical Psychology, University of Lyon 2, Lyon, France

**Keywords:** borderline personality disorder, normal personality, neurotic personality, inventory of personality organisation, elderly patients (participants), nursing homes, long-term care hospital, department of geriatric medicine

## Introduction

This dataset was built with the purpose of clarifying the diagnosis of borderline personality disorder (BPD) in ageing. This clarification is necessary for two reasons. First, dimensional approaches have taken an important standpoint as an alternative model for the diagnosis of personality disorders. This approach is fine grained and more sensitive than the categorical model (First, 2006). Developed in connexion with the theories of personality, the dimensional approach makes it possible to refine psychiatric practise (First, 2005; De Fruyt et al., 2017). In contrast, this model is criticised for its difficulty of application and conceptualisation, as well as for its validity (Chaine and Guelfi, 1999). The significant variability of the profiles isolated only on the basis of the *Diagnostic and Statistical Manual of Mental Disorders*, 5th Edition (DSM-5, American Psychiatric Association, 2013), requires a combined use of tools to assess the actual presence of BPD, because it is common to find a potential co-morbidity with other disorders such as depression, anxiety or post-traumatic stress disorder, sometimes extreme (Frost et al., 2018; Gunderson et al., 2018). Some argue that it might lead to a relative unreliability of the categorical diagnosis (Hörz-Sagstetter et al., 2018). Second, this clarification helps typically to solve the diagnostic difficulties in the assessment of personality disorders in ageing people (Mattar and Khan, 2017). It has been proposed that the specific conditions of expression of BPD in the elderly population differ significantly from their expression in middle-aged adults (Beatson et al., 2016) even though its prevalence in institutions makes it the most common personality disorder (Ellison et al., 2018). Nevertheless, the correct assessment of borderline personality organisation (PO) remains a central concern that affects the clinician in the choice and planning of the therapeutic treatment (Gunderson et al., 2018) as well as in its management (Gordon et al., 2019).

This dataset focuses on the dimensions of personality theorised by Kernberg (Kernberg, 1975; Kernberg and Caligor, 2005) and uses a related test: The Inventory of Personality Organisation (IPO) (Kernberg and Clarkin, 1995). Kernberg's theoretical model situates the borderline personality on a continuum between normal and pathological where the different organisations (neurotic, borderline and psychotic) correspond to specific symptomatological and aetiological criteria. The nature and severity of the disturbances experienced by the participants are assessed according to the level of integration of the identity, the mobilised defence mechanisms and the nature of the reality event. The main difficulty in terms of assessment lies in defining the cut-off scores on the normal–neurotic–borderline continuum.

This dataset was built with 444 responses to a self-assessment questionnaire that were collected using the validated French form of the IPO (Biberdzic, 2017). Data collected come from men and women over 65 years old. Items were rated on a 5-point Likert scale ranging from “never true” to “always true.” Raw data were the sum of the items corresponding to IPO's scales. Age, gender, level of education, and co-morbidities were also indicated.

## Materials and Methods

### Design

This research used a cross-sectional design. The research protocol was developed in collaboration with the Universities of Angers and Lyon 2; and as it was considered as routine by the institutions, it did not have to be submitted to the ethics committee. The protocol has been ethically and methodologically validated and was carried out with the approval of the Gerontology Medical Unit of Lucien Hussel Hospital Center. The research population was evaluated in two French nursing homes, one department of geriatric medicine and one long-term care hospital. The establishments concerned authorised this research. The clinical psychologist in charge of the study collected these data in the geriatric establishments. Data were collected from April 1, 2018, to March 31, 2020. Free and informed consent to participate in the research was requested and signed, in accordance with Code de déontologie des psychologues (1996, 2012). Research participants were men and women over 65 years of age. All data have been anonymised.

To focus on the normal-borderline PO continuum, four major exclusion criteria were defined:

1) major cognitive impairment or dementia,
2) psychotic symptomatology/psychotic PO and major organic or somatic disorder, which can be a source of significant bias in clinical dynamics and testing,
3) diagnosis of BPD and/or psychiatric history, and
4) disabling visual or praxic impairment to testing and major somatic or psychiatric crisis, or any similar disability that may prevent participants from completing the self-assessment questionnaire.

The sample could present minor cognitive disorders that did not significantly affect the participants' thinking and cognitive abilities. Participants needed to have the ability to attend to the survey task. The exclusion criteria were clinically assessed by the psychologist and by the establishment's medical staff before nominating prospective participants for the research.

Socio-demographic information was collected (age, gender, and level of education) to ensure the internal and external validity of the sample. Several tests validated in French were then carried out: the Mini-Mental State Examination (MMSE; Kalafat et al., 2003), the brief version of the Geriatric Depression Scale (GDS; Clément et al., 1997) and the Generalised Anxiety Disorder-7 (GAD-7; Micolaud-Franchi et al., 2016). Finally, we proposed an assessment of PO with the IPO completed by the participants. Participants had to fill in the French form of the international scale of the IPO (Biberdzic, 2017). We remained available if necessary to clarify any misunderstandings or offer any help required.

Socio-demographic information and the test results are presented in Table 1. The resulting dataset corresponds to the participants' responses (see Supplementary Material).

**Table 1:** Descriptive statistics of the sample.

	**Cluster 1**		**Cluster 2**		**Cluster 3**		**Total**
	**(*****n*** **=** **165, 37.1%)**		**(*****n*** **=** **173, 39.0%)**		**(*****n*** **=** **106, 23.9%)**		**(*n* = 444)**
**Level of education**							
Primary	110 (66.7%)		119 (68.8%)		70 (66.0%)		299
Secondary	44 (26.7%)		45 (26.0%)		29 (27.4%)		118
Tertiary	11 (6.6%)		9 (5.2%)		7 (6.6%)		27
MMSE	26.27 (SD = 1.7)		26.03 (SD = 1.6)		26.10 (1.4)		26.13 (SD = 1.6)
MMSE/year/education	65–69		70–74		75–79		80+
Primary	26.4 (SD = 0.9)		25.8 (SD = 0.7)		25.7 (SD = 1.0)		24.8 (SD = 1.0)
Secondary	28.8 (SD = 0.8)		27.8 (SD = 0.8)		26.7 (SD = 1.4)		25.9 (SD = 1.1)
Tertiary	29.2 (SD = 1.0)		29.0 (SD = 0.7)		29.0 (SD = 0)		28.3 (SD = 1.0)
**Co-morbidities**							
Depression (GDS-15 ≥ 5)	33 (20.0%)		60 (34.7%)		71 (66.9%)		164 (36.9%)
GDS-15 <5	132 (80.0%)		113 (65.3%)		35 (33.0%)		
GDS-15 = 5–10	27 (16.4%)		51 (29.5%)		53 (50.0%)		
GDS-15 > 10	6 (3.6%)		9 (5.2%)		18 (17.0%)		
Anxiety (GAD-7 > 8)	11 (6.6%)		23 (13.3%)		40 (37.7%)		74 (16.6%)
Minimal anxiety (<5)	100 (60.6%)		74 (42.8%)		36 (33.9%)		
Mild anxiety (5–9)	57 (34.5%)		82 (47.4%)		38 (35.8%)		
Moderate anxiety (10–14)	8 (4.8%)		17 (9.8%)		30 (28.3%)		
Severe anxiety (≥15)	0		0		2 (1.9%)		
Anxiety + Depression	10 (6.0%)		21 (12.1%)		38 (35.8%)		69 (15.5%)
**IPO scales**	**Mean**	**SD**	**Mean**	**SD**	**Mean**	**SD**	
PD	22.3	7.9	41.5	9.8	61.8	9.6	
ID	52.7	8.7	74.2	10.2	95.6	9.3	
RT	28.4	6.6	36.9	7.3	56.8	8.9	
A	23.8	6.2	39	10.3	56.1	9.1	
MV	14.3	6	28.5	6.4	38.4	3.4	

*MMSE, Mini-Mental State Examination; GDS-15, Geriatric Depression Scale-15; GAD-7, Generalised Anxiety Disorder-7; IPO, Inventory of Personality Organisation; PD, primitive defences; ID, identity diffusion; RT, reality testing; A, aggression; MV, moral values*.

### Material

The MMSE is a 30-point questionnaire that is used extensively in clinical and research settings to measure cognitive impairment. It is commonly used in medicine and allied health to screen for dementia. Kalafat et al. (2003) developed standards for the general French population. A score of <24 indicates a cognitive impairment probably associated with functional impairment. However, for people aged over 80 with a low socio-cultural level, a score below 23 is considered to be an index of cognitive impairment. The further the score is from 23, the greater the deficit.

The short form of the 15-item GDS (GDS-15) is a self-report measure of depression in older adults. It is the French reference questionnaire for the assessment of depression in the elderly (Clément et al., 1997). A score of <5 corresponds to an absence of depression. A score between ≥5 and ≤ 10 indicates mild to moderate depression. For a score > 10, the depression is severe.

The GAD-7 (Micolaud-Franchi et al., 2016) is a self-report scale used for screening, diagnosis and severity assessment of anxiety disorder. It is a seven-item questionnaire with a total score of 21. Each item is rated according to the Likert scale from 0 to 3. Scores of 5, 10, and 15 represent cut-off points for mild, moderate and severe anxiety, respectively. A score > 8 indicates a very high probability of having an anxiety disorder.

The IPO is an 83-item self-assessment questionnaire. It has been the subject of many publications and has been translated into several languages (Lenzenweger et al., 2001). The questionnaire consists of five scales, which are based on Otto Kernberg's theory of personality dimensions (Kernberg and Clarkin, 1995; Lenzenweger et al., 2001). Items are rated on a 5-point Likert scale ranging from “never true” to “always true.”

The first three scales assess the three main dimensions of Kernberg's PO model, i.e., the degree of maturity of defence mechanisms (whether or not emotional regulation is allowed), reality testing (particularly the ability to maintain contact with reality) and identity diffusion (a poorly integrated identity vs. an ability to develop a nuanced, complex and stable perception of oneself and of others). Kernberg added three secondary criteria to the three basic ones presented above, in order to adjust his nosographic approach. The first criterion is the quality of aggression. It assesses whether the subject's internal life and external behaviour are dominated by aggression and evaluates the defences used against it. The second criterion is the quality of moral values. It assesses how the subject has internalised stable values and morals and how this affects his/her internal experience and guides his/her behaviour. The third criterion is the quality of the predominant object relation (i.e., the nature and the stability of intimate and interpersonal relationships that a person has with others).

The five scales comprising the IPO are as follows: the Primitive Defences scale (PD = 16 items), the Identity Diffusion scale (ID = 21 items), the Impaired Reality Testing scale (RT = 20 items), the Aggression scale (A = 18 items) and the Moral Values scale (MV = 8 items). These five scales make it possible to assess different “personality organisations.” Kernberg's model defines the organisation of personality as a rather stable structure, which is part of a continuum from normal to pathological personality. The level of personality organisation is measured on the basis of the evaluation of the five scales comprising the IPO. In Kernberg's model, three main levels of organisation are identified: normal-neurotic, borderline and psychotic organisation. According to Kernberg, differences between normal and neurotic organisation are mainly due to changes in the use of the PD. These changes are more quantitative than qualitative (Agnieszka, 2015). For borderline and psychotic personality organisations, the differences are also quantitative, but variations produce qualitative changes. The psychotic personality organisation is characterised by impairments in all five dimensions. Psychotic personality organisation is excluded from this research because of its diagnostic specificity, questions regarding differential diagnosis and its specific manifestations in the elderly (Beatson et al., 2019). Data focus only on the normal-borderline continuum.

### Participants

A total of 444 responses to all questionnaires were collected from participants in three locations. The collected data came from men and women over 65 years old [mean age 77.8 ± 9.20 standard deviation (SD), range 65–97 years]. Of the total sample, 211 of 444 participants were male (mean age 77.7 ± 9.00 SD, range 65–97 years) and 233 were female (mean age 78.0 ± 9.00 SD, range 65–96 years).

Concerning co-morbidities (see Table 1), 36.9% of participants had depressive symptoms. Participants with borderline personality organisation had more severe depressive symptoms (18) than neurotic personality organisation (9) or normal personality (6). There were 16.6% of participants with anxiety symptoms. Also, participants with borderline personality organisation had more moderate-to-severe anxiety symptoms (32) than neurotic personality organisation (17) or normal personality (8). Anxiety associated with depression was more present in borderline personality organisation (38) than neurotic personality organisation and normal personality with 21 and 10 participants, respectively.

## Data Analysis

Table 1 presents descriptive statistics of the sample and defines different personality groups after an agglomerative hierarchical clustering (AHC) analysis using an aggregation method with complete linkage and automatic truncation by entropy. Table 2 corresponds to cut-off scores for each personality organisation. Several factors were compared: sensitivity, specificity, positive and negative predictive values, positive and negative likelihood ratios and Youden's index (reduced false positives and false negatives).

**Table 2:** Cut-off scores and precision indexes for the normal and borderline personality organisations on the IPO scales for older adults (age ≥ 65 years).

**Subscales**	**Criterion**	**Sensitivity**	**Specificity**	**Youden's index**	**+LR**	**–LR**	**+PV**	**–PV**
**Normal PO**								
PD	≤ 31	0.871	0.907	0.778	9.145	0.142	84.5	92.4
ID	≤ 46	0.903	0.91	0.813	10.078	0.107	85.6	94.1
RT	≤ 33	0.859	0.786	0.645	4.022	0.179	70	90.6
A	≤ 29	0.81	0.94	0.75	13.386	0.202	88.6	89.5
MV	≤ 21	0.902	0.915	0.817	10.559	0.107	86	94.1
**Borderline PO**								
PD	≥47	0.943	0.861	0.804	6.784	0.066	68	98
ID	≥59	0.972	0.867	0.839	7.299	0.033	69.6	99
RT	≥47	0.887	0.938	0.825	14.273	0.121	81.7	96.4
A	≥44	0.953	0.882	0.835	8.051	0.054	71.6	98.3
MV	≥34	0.906	0.876	0.782	7.288	0.108	69.6	96.7

*IPO, Inventory of Personality Organisation; PO, personality organisation; PD, primitive defences; ID, identity diffusion; RT, reality testing; A, aggression; MV, moral values; +/–LR, positive/negative likelihood ratios; +/–PV, positive/negative predictive values*.

The data considered as non-IPO according to level of education, MMSE, GDS-15, and GAD-7 seemed to validate its consistency (Table 1). The data collected were consistent with the known data on the level of education of the French population and variations according to gender (OCDE, 1999, 2020), i.e., 59% of the population had an education level below secondary, 26% had a secondary level and 6% had a higher level. The relationship between the level of education and the results obtained in the MMSE is verified (Bravo and Hébert, 1997, see Table 1). Assessment of depression with GDS-15 provided similar data to other studies (e.g., Limosin et al., 2015), i.e., up to 40% depression in nursing homes, 30% of which was severe depression (Thomas and Hazif-Thomas, 2013). Regarding the assessment of anxiety, results were consistent with those observed in a similar population (Creighton et al., 2016), up to 20% of the estimated population. The relationship between anxiodepressive symptoms that were specific to the population assessed (Ulbricht et al., 2019) is also validated: up to 25.2%. The accumulation of these different disorders increased with their severity (Smalbrugge et al., 2005), which our sample also demonstrated (*r* = 0.736, *p* < 0.001).

IPO had an adequate internal consistency in the population tested (Cronbach's alpha = 0.934). Respondents were grouped on the basis of AHC using an aggregation method with complete linkage and automatic truncation by entropy. Three clusters were identified. They correspond closely to the clusters identified by Hörz-Sagstetter et al. (2018):

- The normal personality organisation (Cluster 1, Table 1) is defined by a very low use of primitive defence mechanisms (PD), a low level of identity diffusion (ID) corresponding to a “consolidated identity,” low impairments to the reality testing (RT), a low level of aggression (A) and a very low level of disruption of the moral value system (MV).
- The neurotic personality organisation (Cluster 2, Table 1) is defined by a moderate use of primitive defence mechanisms (PD), a medium level of identity diffusion (ID), moderate impairments to the reality testing (RT), a moderate level of aggression (A) and a low level of disruption of the moral value system (MV).
- The borderline personality organisation (Cluster 3, Table 1) is characterised by a high use (=predominant) of primitive defence mechanisms (PD); a high level of identity diffusion (ID), which corresponds to a marked identity diffusion; moderate impairments to the reality testing (RT), i.e., a broadly intact reality testing; a moderate level of aggression (A); and a moderate level of disruption of the moral value system (MV).

The borderline personality organisation represented 23.8% of the sample. Its prevalence was consistent with what has been observed internationally (Valdivieso-Jiménez, 2018). It was also consistent with data previously collected in the French population (Guelfi et al., 2011). Indeed, its prevalence was between 18 and 42.7% in a clinical population. These results clarified the close link already observed between borderline personality organisation and depression in the elderly population living in geriatric institutions (Beatson and Rao, 2013; Beatson et al., 2016). The data collected also make it possible to validate the over-representation of anxiety elements in the borderline group in accordance with what has already been observed in the elderly (Hellwig and Domschke, 2019). Cluster A and B personality disorders such as schizotypal, borderline and narcissistic personality disorders were identified as predictors of incident panic disorder, social phobia and GAD, respectively, in a nationally representative sample of 8012 community-dwelling adults aged ≥60 years interviewed twice over a period of 3 years (Chou et al., 2011). Regarding depression, ANOVA showed a difference between at least two of the three clusters (*p* < 0.001). The *post-hoc* Tukey test showed a significant difference between Clusters 1 and 3 and groups 2 and 3 (*p* < 0.001). Cluster 3 had a significantly higher (more pathological) depression score than the other two groups. These results were identical for anxiety where group 3 had a significantly higher score (therefore more pathological) than the other two groups (*p* < 0.001; Tukey). Regarding the results obtained by the participants in the MMSE, a significant difference concerning study level (*p* < 0.001) was found. This holds true for all three modalities (*p* < 0.001; Tukey). For the clusters, no significant difference could be observed (*p* = 0.385). Regarding the results of the IPO, there was a significant difference between the clusters on the PD, ID, and RT subscales (*p* < 0.001; *p* < 0.001 for Tukey test). On these three dimensions, the borderline group obtained a significantly higher score than did the other groups. This difference is also verified for Cluster 2 compared with Cluster 1. These data supported the internal validity of the sample and the subgroups.

Cut-off scores for the diagnosis of borderline personality organisation were defined (Table 2) by the receiver operating characteristics curve (ROC curve) and area under the curve (AUC). Thus, according to the normal–pathological continuum, a participant can present with borderline personality organisation or not (Clusters 1–3, Table 1). ROC curves evaluate the performance of a binary classifier. They display the contrast between the two most distinct groups on the normal–pathological continuum, i.e., normal vs. borderline personality organisation. AUCs for the normal personality organisation range from 0.888 (95% CI 0.855–0.916) for the Reality Testing (RT) scale to 0.959 (95% CI 0.936–0.975) for the Moral Values (MV) scale. Concerning the borderline personality organisation, AUCs range from 0.943 (95% CI 0.917–0.962) on the Aggression (A) scale to 0.972 (95% CI 0.952–0.985) on the Identity Diffusion (ID) scale (Table 2).

To define the cut-off scores for each personality organisation, the following factors were compared on all scales: sensitivity, specificity, positive and negative predictive values, positive and negative likelihood ratios and Youden's index (Table 2). For each element, the higher the value, the more precise the discrimination and therefore the more effective the test. The results of the evaluation of the ROC curves (i.e., assessment of the performance of a binary classifier) produced cut-off scores for normal and borderline groups.

The precision of the classification indexes (sensitivity, specificity, positive and negative predictive values, positive and negative likelihood ratios and Youden index) revealed in these groups of respondents that (Table 2):

- Scores ≤ 31 for the Primitive Defences (PD), 46 for the Identity Diffusion (ID), 33 for the Reality Testing (RT), 29 for the Aggression (A) and 21 for the Moral Values (MV) are representative of the normal organisation of the personality.
- Scores ≥ 47 for Primitive Defences (PD), 59 for Identity Diffusion (ID), 47 for Reality Testing (RT), 44 for Aggression (A) and 34 for Moral Values (MV) are representative of the borderline personality organisation.

The binary classification that resulted from the ROC curves and the AUCs produced cut-off scores for the normal and borderline personality organisations (Table 2), the precision indexes having been evaluated for these two organisations. Consequently, cut-off scores of the third personality organisation—neurotic—are defined between the cut-off scores of normal personality organisation and borderline personality organisation:

- Scores between 32 and 46 for Primary Defences (PD), 46 and 58 for Identity Diffusion (ID), 34 and 46 for Reality Testing (RT), 30 and 43 for Aggression (A), and 22 to 33 for Moral Values (MV) are representative of a neurotic personality organisation.

## Data Usage and Application

This dataset is useful because its analysis establishes cut-off scores, which facilitate the diagnosis of borderline personality organisation. It helps to distinguish normal from neurotic personality organisations:

- These data show an effective alternative to the diagnosis of BPD in ageing only based on DSM-5, the latter having many limitations (Beatson et al., 2016).
- These data can be easily used as part of a gerontological assessment. They can be used to identify personality organisation (neurotic or borderline). Also, borderline personality organisation can be assessed on a normal–pathological continuum.
- These data can be analysed as part of a lifespan approach to borderline personality organisation, integrated with data relating to the IPO in participants over 65 years of age and included in meta-analyses.
- These data provide additional correlated information on rates of depression and anxiety in the elderly population in geriatric institutions.

## Limitations

A comprehensive assessment and an independent diagnosis of BPD could have been done by an experienced psychiatrist regardless of the existence of screening/diagnostic tools. Our data tend to indicate typical profiles (normal, neurotic and borderline) consistent with Kernberg's theory (Hörz-Sagstetter et al., 2018). However, future research should focus on comparing these data with those obtained in the broader community. It will also be important to study the challenges of using the IPO in ageing by comparing it with other tools. This will make it possible to assess and determine the structural organisation of the individual personality.

## Data Availability Statement

The datasets presented in this study can be found in online repositories. The names of the repository/repositories and accession number(s) can be found below: http://dx.doi.org/10.17632/zc47xbw2kk.2.

## Ethics Statement

Ethical review and approval was not required for the study on human participants in accordance with the local legislation and institutional requirements. The patients/participants provided their written informed consent to participate in this study.

## Author Contributions

FR-G, LP, and JL designed the study, analysed data, and wrote the draught of the manuscript. LP and JL collected data. FR-G reviewed the manuscript for important intellectual content. All authors listed have made a substantial contribution to the conception, development of methodological approach and interpretation of results, and approved it for publication.

## Conflict of Interest

The authors declare that the research was conducted in the absence of any commercial or financial relationships that could be construed as a potential conflict of interest.
